# GDF15 expression in glioma is associated with malignant progression, immune microenvironment, and serves as a prognostic factor

**DOI:** 10.1111/cns.13749

**Published:** 2021-10-25

**Authors:** Longbin Guo, Yulei Chen, Shushu Hu, Lianxuan Gao, Nan Tang, Rongping Liu, Yue Qin, Chen Ren, Shasha Du

**Affiliations:** ^1^ Department of Radiation Oncology Nanfang Hospital Southern Medical University Guangzhou China

**Keywords:** GDF15, glioma, biomarker, Immune, prognosis

## Abstract

**Aims:**

Growth differentiation factor 15 (GDF15) is involved in lots of crucial inflammatory and immune response. The clinical and immune features for GDF15 in glioma have not been specifically investigated so far.

**Methods:**

Gene expression profiles obtained from public glioma datasets were used to explore the biological function of GDF15 and its impact on immune microenvironment. Interference with GDF15 in several glioma cell lines to verify its functions in vitro. Survival data were used for the survival analysis and establishment of a nomogram predictive model.

**Results:**

GDF15 was up‐regulated in various malignant phenotypes of glioma. Function analysis and in vitro experiments revealed that GDF15 was associated with malignant progression and NF‐κB pathway. GDF15 was closely correlated to inflammatory response, infiltrating immune cells, and immune checkpoint molecules, especially in lower grade glioma (LGG). High expression level of GDF15 predicted poor survival in LGG, while the effect on glioblastoma (GBM) was not significant. A nomogram predictive model combining GDF15 and other prognostic factors was constructed and showed ideal predictive performance.

**Conclusions:**

GDF15 could serve as an interesting prognostic biomarker for LGG. Regulating the expression of GDF15 may help solve the dilemma of immunotherapy in glioma.

## Graphical abstract

In this study, we conducted in‐depth analysis of gene expression profiles and in vitro experiments, and found that GDF15 is closely related to the malignant progression, immune microenvironment, and prognosis of glioma. GDF15 could be served as a novel prognostic and immune biomarker for glioma.

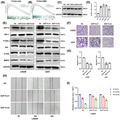

## 1 BACKGROUND

Glioma is the most common primary brain tumor of the central nervous system.^1^ Despite receiving standard therapeutic regimens, the prognosis of glioma is still unsatisfactory.^2^ Identification of key driven factors governing glioma progression could not only provide in‐depth understanding of the disease but also may predict prognosis and serve as target for precise treatment. Meanwhile, the success of immunotherapy in solid tumors has brought new opportunities for glioma treatment.^3^ However, the current relevant clinical trials in glioma have not shown encouraging results.^4^, ^5^ The unique and complex immune microenvironment of glioma is the crucial obstacle to immunotherapy.^6^ Thus, it is also urgent to figure out the potential mechanism and factors that affect the immune microenvironment of glioma, which may help improve efficacy of immunotherapy.

Growth differentiation factor 15 (GDF15), a divergent member of the transforming growth factor‐beta (TGF‐β) superfamily, has received appreciable attention in the last two decades for its multiple key role in several diseases, including obesity, cachexia, and cardiovascular disease.^7^, ^8^, ^9^ Nowadays, GDF15 is regarded as a marker of oxidative stress and an inflammation‐induced central mediator of tissue tolerance.^8^, ^10^ Recently, several studies have demonstrated the close links between GDF15 and the development of cancers.^11^, ^12^, ^13^ GDF15 performs pleiotropic functions in tumor progression, acting either as suppressor or as promoter in early and late stages of tumors, respectively.^14^, ^15^ It has been shown that GDF15 may influence glioma cell invasion, and elevated level of GDF15 in the cerebrospinal fluid is associated with worse outcome of GBM patients.^16^, ^17^ However, the clinical and immune features for GDF15 in glioma have not been specifically investigated so far.

In this study, we found that GDF15 correlated with malignant progression in glioma using clinical and transcriptome (RNA‐seq) data from two large glioma datasets, including The Cancer Genome Atlas (TCGA) and Chinese Glioma Genome Atlas (CGGA) datasets. The biological characteristics and functions of GDF15 were identified based on RNA‐seq and verified in cell experiments. Besides, we showed that GDF15 closely related to immune response, immune infiltration cells, and immune checkpoint molecules, especially in LGG, highlighting its important role in immune microenvironment. Survival analysis revealed GDF15 predicted poor survival for LGG and could be served as a novel prognostic biomarker.

## 2 MATERIALS AND METHODS

### 2.1 Data collection

Gene expression and glioma survival data in TCGA were obtained from GlioVis (http://gliovis.bioinfo.cnio.es/), and CGGA were obtained from http://www.cgga.org.cn/. A total of 1362 samples of RNA‐seq data were included. RNA‐seq data from different datasets were uniformly log2 transformed. Samples without complete gene expression profiles were first excluded.

### 2.2 Cell culture

Human astrocytes (HA) cell line was obtained from ScienCell Research Laboratories. Human glioma cell lines such as U87, T98G, LN229, and U251 were purchased from Chinese Academy of Sciences Cell Bank. Cell lines were cultured in DMEM with 10% fetal bovine serum and 1% penicillin/streptomycin. All cells were maintained in an incubator containing 5% CO2 at 37°C. Cells were passaged when reaching 90% confluence.

### 2.3 Small interference RNA (siRNA) transfection and validation

According to the product instructions (Ruibo company), siRNA was transfected at an siRNA concentration of 100nM. Twenty‐four hours after transfection, medium without siRNA was added to the cells. Western blot analysis was performed with rabbit anti‐GDF15 antibody (1:1000, cat. no.8479; CST). Goat anti‐rabbit IgG‐HRP (1:5000, ab6721, Abcam) was used as secondary antibodies and GAPDH (1:1000, cat. no.5174; CST) was used for loading control. Total RNA from cells was extracted using TRIzol reagent (Invitrogen). The Reverse Transcription System kit (Takara Bio, Inc.) was used to perform RNA reverse transcription reactions. The real‐time (RT) quantitative polymerase chain reaction (PCR) was performed using the SYBR Green real‐time PCR kit (Takara). The forward and reverse primers sequences (5'‐3') of GDF15 and β‐actin were given in the following: GDF15‐Forward, ACCTGCACCTGCGTATCTCT; GDF15‐Reverse, CGGACGAAGATTCTGCCAG; β‐actin‐Forward, TGGCACCCAGCACAATGAA; and β‐actin‐Reverse, CTAAGTCATAGTCCGCCTAGAAGCA. The relative quantitative data of mRNAs were normalized to β‐actin and quantified using the 2^−ΔΔCt^ method.

### 2.4 Western blot

Cells were lysed with protein lysis buffer containing a cocktail of protease and phosphatase inhibitors (Roche). Total protein was extracted by centrifugation at 15,000*g* at 4°C for 30 min. Protein were transferred to membranes following SDS‐PAGE, and the membrane was blocked with 5% non‐fat dry milk for 1h. All primary antibodies were used at a 1:1000 dilution at 4°C for 24 h. Antibodies used are given in the following: IκBα Mouse Antibody (1:1000, cat. no.4814; CST), Phospho‐IκBα Rabbit Antibody (1:1000, cat. no.2859; CST), NF‐κB p65 Rabbit Antibody (1:1000, cat. no.8242; CST), Phospho‐NF‐κB p65 Rabbit Antibody (1:1000, cat. no.3033; CST), MMP‐9 Rabbit Antibody (1:1000, cat. no.13667; CST), and GAPDH Rabbit Antibody (1:1000, cat. no.5174; CST). The results were visualized using a chemiluminescence detection system (Bio‐Rad Laboratories, Inc.).

### 2.5 Cell scratch assays

U251 and LN229 cells were seeded in six‐well plates (5 × 104 cells per well) and transfected with GDF15 siRNA or a NC siRNA. When the cell grew to 100% confluency, sterile 200 μl pipette tip was used to scratch and washed three times with PBS to remove the floating cells. The cells were cultured with serum‐free medium and incubated in a 37°C, 5% CO_2_ incubator. Cells were observed by microscopy and photographically recorded (0 h, 24 h, and 48 h cell scratches).

### 2.6 Cell migration assays

Twenty‐four‐well transwell chambers (8 μm pore size, Corning) were used for the assay. After 24 h transfection with siRNA, 1 × 105 Cells in 100 μl serum free medium were seeded in upper chambers. 600 μl medium containing 10% FBS was added to the lower chamber. After 16 h, the migration cells were fixed using 4% paraformaldehyde, stained with 0.05% crystal violet, and counted from five random fields.

### 2.7 Bioinformatics analysis

Wilcoxon test was used to investigate the expression pattern of GDF15 in various subtypes and molecular characteristics of glioma. UCSCXena (https://xenabrowser.net/) was used to analyze the relationship between GDF15 and the copy number of EGFR, PTEN, chromosomes 7 and 10 in TCGA. Gene Set Enrichment Analysis (GSEA)^18^ conducted using clusterProfiler package^19^ was used to identify biological function. Seven immune metagene clusters referenced to a previous study^20^ were established to represent various types of inflammatory response. The expression heatmaps were generated using pheatmap package. Gene Sets Variation Analysis (GSVA)^21^ and corrgram packages (https://github.com/kwstat/corrgram) were used to determine the enrichment status of inflammatory response metagene clusters. Relatively abundance of tumor infiltrating immune cells was evaluated by ImSig package.^22^ TIMER (https://cistrome.shinyapps.io/timer/) was employed to correct the effect of tumor purity on the expression of genes and performed correlation analysis.^23^ The correlation between GDF15 and immune checkpoint molecules was evaluated by Pearson's correlation test and converted to chord diagram using circlize package (https://github.com/jokergoo/circlize).

### 2.8 Survival analysis and construction of predictive model

Patients with missing important clinical data were further eliminated here. A total of 588 patients from TCGA and 508 patients from CGGA were selected (Table S1). Survminer package (https://github.com/kassambara/survminer) was used for Kaplan–Meier survival analysis, and log‐rank test was used to evaluate the difference between survival curves. Univariate and multivariate Cox regression analyses were used to identify the independent prognostic factors for glioma. The 5 years survival rate was selected as a representative to construct the nomogram using hdnom package (https://github.com/nanxstats/hdnom/). A risk classification system was subsequently developed based on the nomogram.

### 2.9 Statistical analysis

R language (version 3.6.1), GraphPad Prism 7, and SPSS 17.0 (SPSS, Inc.) were used for the statistical analysis and generating figures. For data from cell experiments, test for normality was performed using Shapiro–Wilk test. Data followed a normal distribution were analyzed via Student's *t*‐test and one‐way ANOVA test. Otherwise, data were analyzed via Mann–Whitney test. The continuous variables were showed as mean ± SD. A two‐sided *p*‐value <0.05 was considered to be statistically significant.

## 3 RESULTS

### 3.1 GDF15 was up‐regulated in malignant phenotypes of glioma

**FIGURE 1:**
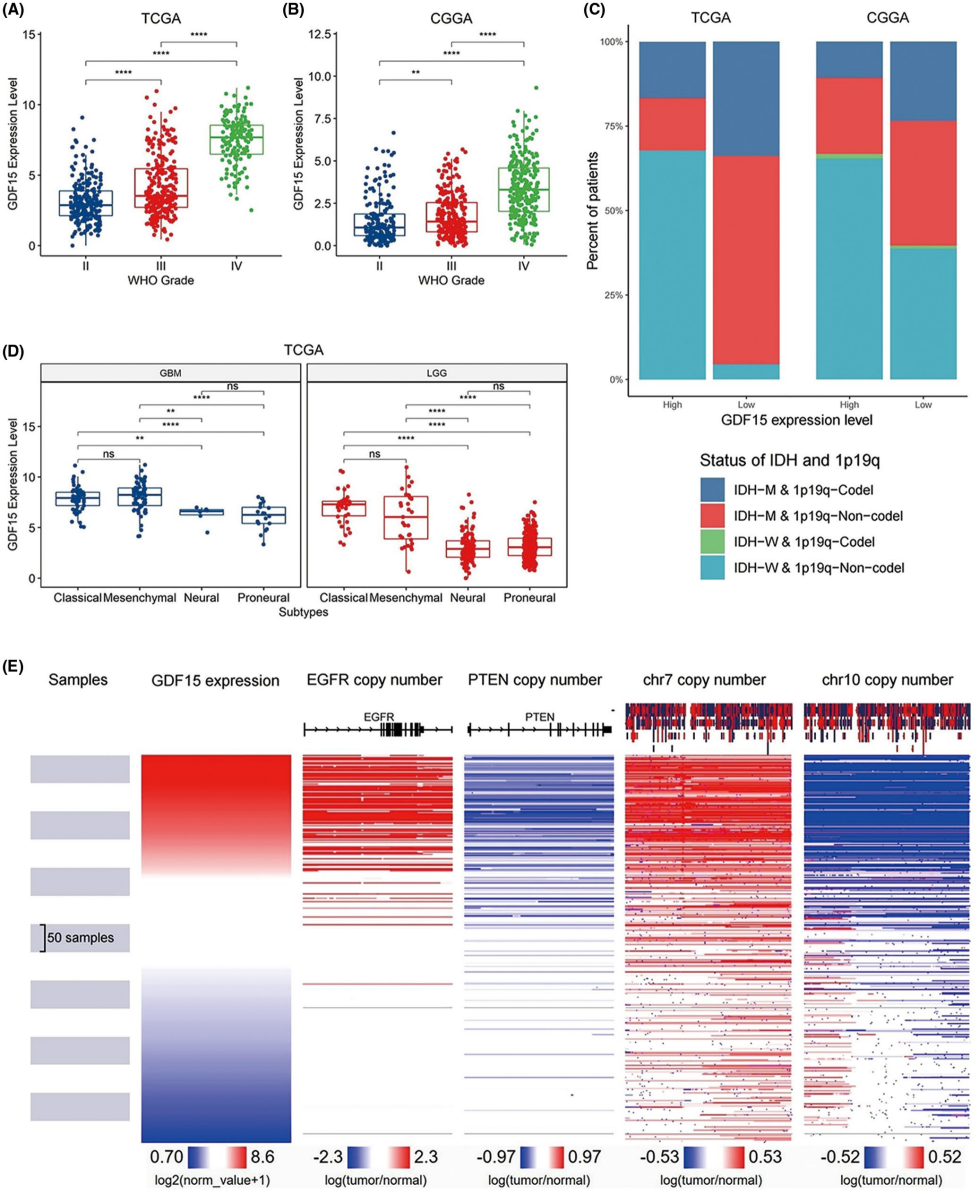
Expression and clinical relevance of GDF15 in glioma. (A, B) The expression pattern of GDF15 in different WHO grades of glioma in TCGA (A) and CGGA (B) datasets. (C) The proportion distribution of patients with different status of IDH and 1p19q in high‐ and low‐GDF15 expression groups in TCGA and CGGA datasets. (D) The expression pattern of GDF15 in different subtypes of glioma in TCGA dataset. (E) The relationship between GDF15 expression and the copy number of EGFR, PTEN, chromosomes 7 and 10 in TCGA dataset. M, mutant. W, wildtype. Codel, co‐deletion. Non‐codel, non‐co‐deletion. *p*‐value significant codes: **** ≤0.0001 < *** ≤0.001 < ** ≤0.01 < * ≤0.05. ns, not significant

We first compared the GDF15 expression levels across different grades in TCGA and found that GDF15 was positively correlated with tumor grades (Figure 1A). The result was validated in CGGA (Figure 1B). Since the status of IDH and chromosome 1p19q had important influence on glioma, the relationship between GDF15 and them was also analyzed. The results showed that patients with high GDF15 expression had higher proportion of IDH wild‐type and 1p19q non‐co‐deletion, which were all signs of poor prognosis (Figure 1C). Similar results could also be observed after distinguishing patients into LGG and GBM (Figure S1A–D). Subsequently, we found that classical and mesenchymal subtypes with poor prognosis had higher GDF15 expression compared with neural and proneural subtypes (Figure 1D). Moreover, high GDF15 expression was significantly related to EGFR and chromosome 7 copy number amplification, as well as PTEN and chromosome 10 copy number reduction, which were both markers of poor prognosis for glioma (Figure 1E). Collectively, GDF15 was up‐regulated in various malignant phenotypes of glioma, suggesting that it may affect glioma prognosis.

### 3.2 GDF15 affected NF‐κB related pathways and progression in glioma

**FIGURE 2:**
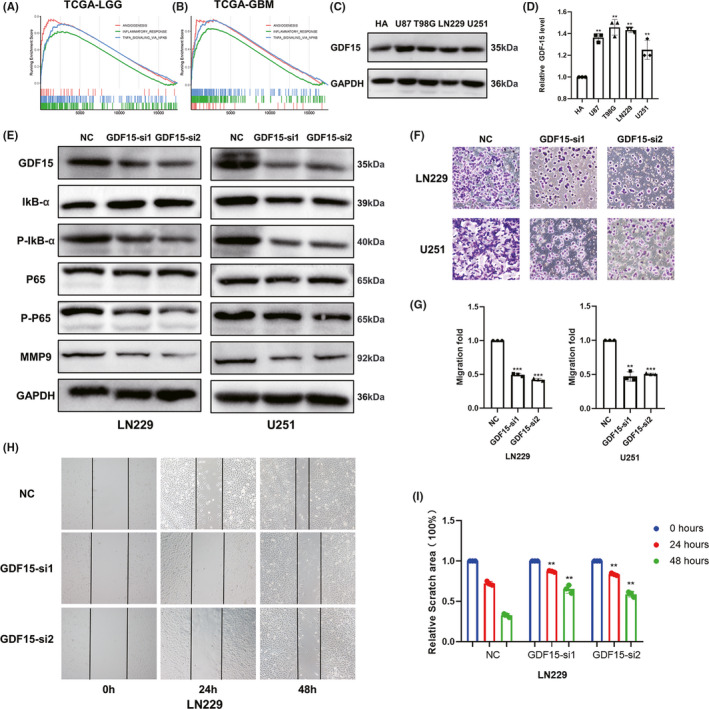
Biological function investigation and in vitro verification of GDF15 in glioma. (A, B) GSEA analysis confirmed several important functions of GDF15 in LGG (A) and GBM (B) of TCGA datasets. (C) The expression of GDF15 protein in the human astrocytes (HA) cell line and glioma cell lines. (D) Quantification of immunoblot results of GDF15 expression in HA cell line and glioma cell lines. (E) The Western blot analysis of NF‐κB signaling and MMP9 protein expression in LN229 and U251 cell lines after transfection with GDF15 siRNA or negative control. (F) Transwell assay of LN229 and U251 cell lines treated with GDF15 siRNA or negative control (magnification, ×100). (G) Quantification of transwell assay of LN229 and U251 cell lines. (H) Cell scratch assay of LN229 cell lines treated with GDF15 siRNA or negative control (magnification, ×100). (I) Quantification of cell scratch assay of LN229 cell lines

To elucidate the biological functions of GDF15 in glioma, we performed GSEA in TCGA and CGGA (Table S2). The results showed that GDF15 was significantly correlated with angiogenesis, inflammatory response, and TNFɑ signaling via NF‐κB (Figure 2A,B, and Figure S2A, B). Experiments in vitro were subsequently performed to confirm our findings. Firstly, we detected GDF15 protein levels in four glioma cell lines and HA cell line by Western blot analysis (Figure 2C,D). We found that the protein levels of GDF15 in glioma cell lines were apparently higher than HA. Then, two kinds of GDF15‐siRNA were transfected to knockdown GDF15 expression in LN229 and U251 (Figure S2C, D). Subsequently, we detected the protein levels of several NF‐κB signaling pathway related biomarkers and MMP9, a hallmark of tumor invasion (Figure 2E, and Figure S2E, F). The results indicated that the protein levels of IκB, P65, and MMP9 had decreased to varying degrees in two glioma cell lines. To further verify the effects of GDF15 on glioma progression, transwell and cell scratch assays were performed (Figure 2F–I, and Figure S2G, H). We found that the migration ability of glioma cell lines was apparently suppressed after transfection with GDF15‐siRNA. Overall, these findings demonstrated that GDF15 affected NF‐κB related pathways in glioma, and played an important role in tumor progression.

### 3.3 Specific relationship between GDF15 and inflammatory response

**FIGURE 3:**
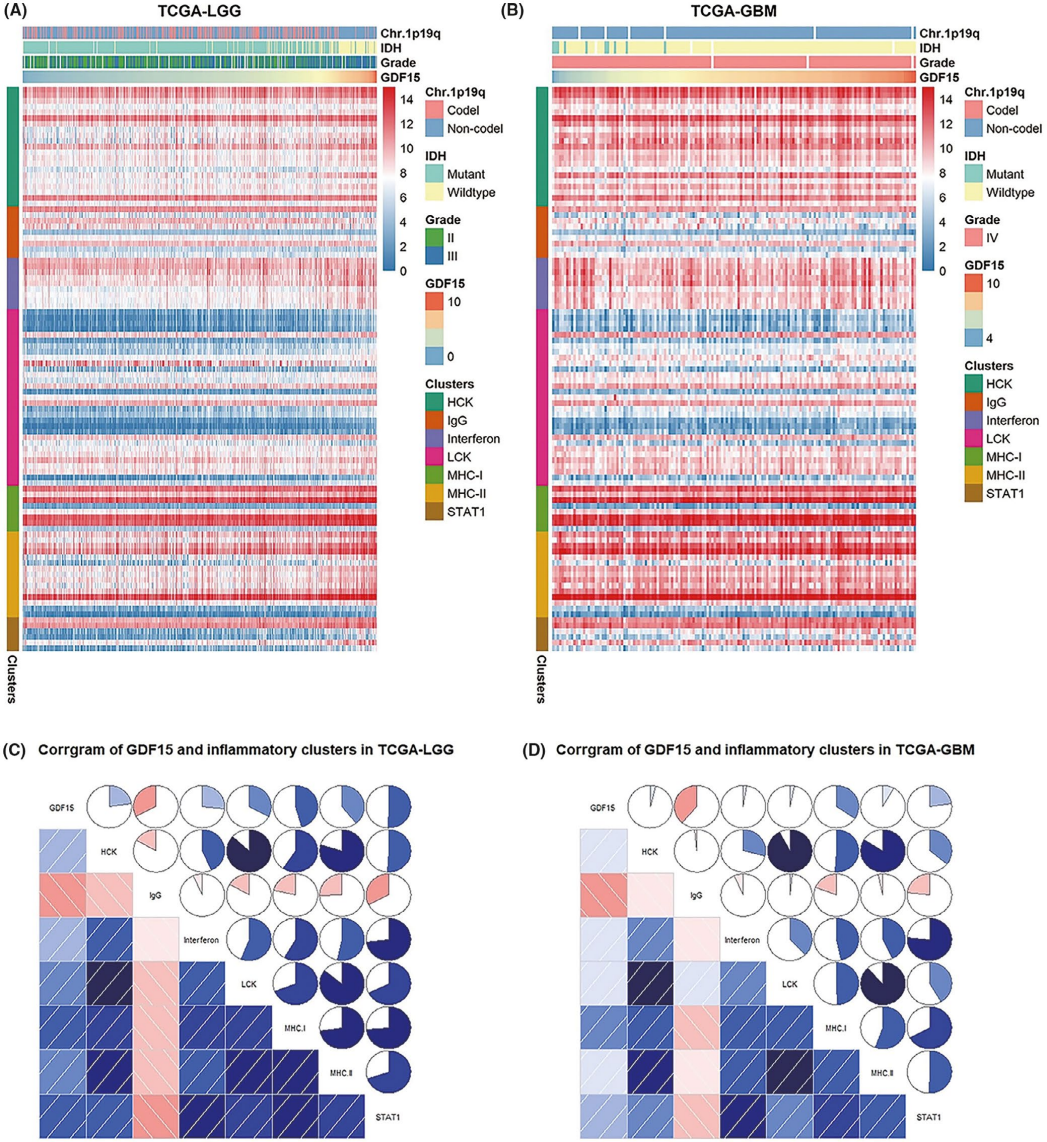
Relationship between GDF15 and inflammatory response in glioma of TCGA dataset. (A, B) Heatmaps showed the disturbance of clinicopathological parameters, GDF15 expression and seven metagene clusters in LGG (A) and GBM (B) of TCGA datasets. (C, D) Corrgrams were generated according to Pearson's correlation values based on GDF15 expression and GSVA enrichment scores for seven metagene clusters. Blue and red represented positive and negative correlations, respectively. Darker colors indicated more significant correlations

Our above finding suggested GDF15 was also closely correlated with inflammatory response. For the sake of studying the specific relationship between GDF15 and inflammatory response, we selected seven metagene clusters, which were previously established to represent various types of inflammatory response.^20^ The expression heatmaps based on LGG cohorts from two datasets showed that GDF15 expression was positively correlated with inflammatory response clusters included HCK, interferon, LCK, MHC‐I, MHC‐II, and STAT1, while only negatively correlated with the IgG (Figure 3A,C, and Figure S3A, C). However, inconsistent results appeared in the GBM cohorts. In TCGA, GDF15 still showed significant correlation with IgG, MHC‐I, and STAT1 clusters, while it was not significantly correlated with other clusters (Figure 3B,D). Meanwhile, GBM cohort in CGGA had the same trend as LGG cohort (Figure S3B, D). Altogether, our findings further indicated GDF15 was closely related to inflammatory response of glioma, especially in LGG.

### 3.4 GDF15 correlated with infiltrating immune cells in glioma microenvironment

**FIGURE 4:**
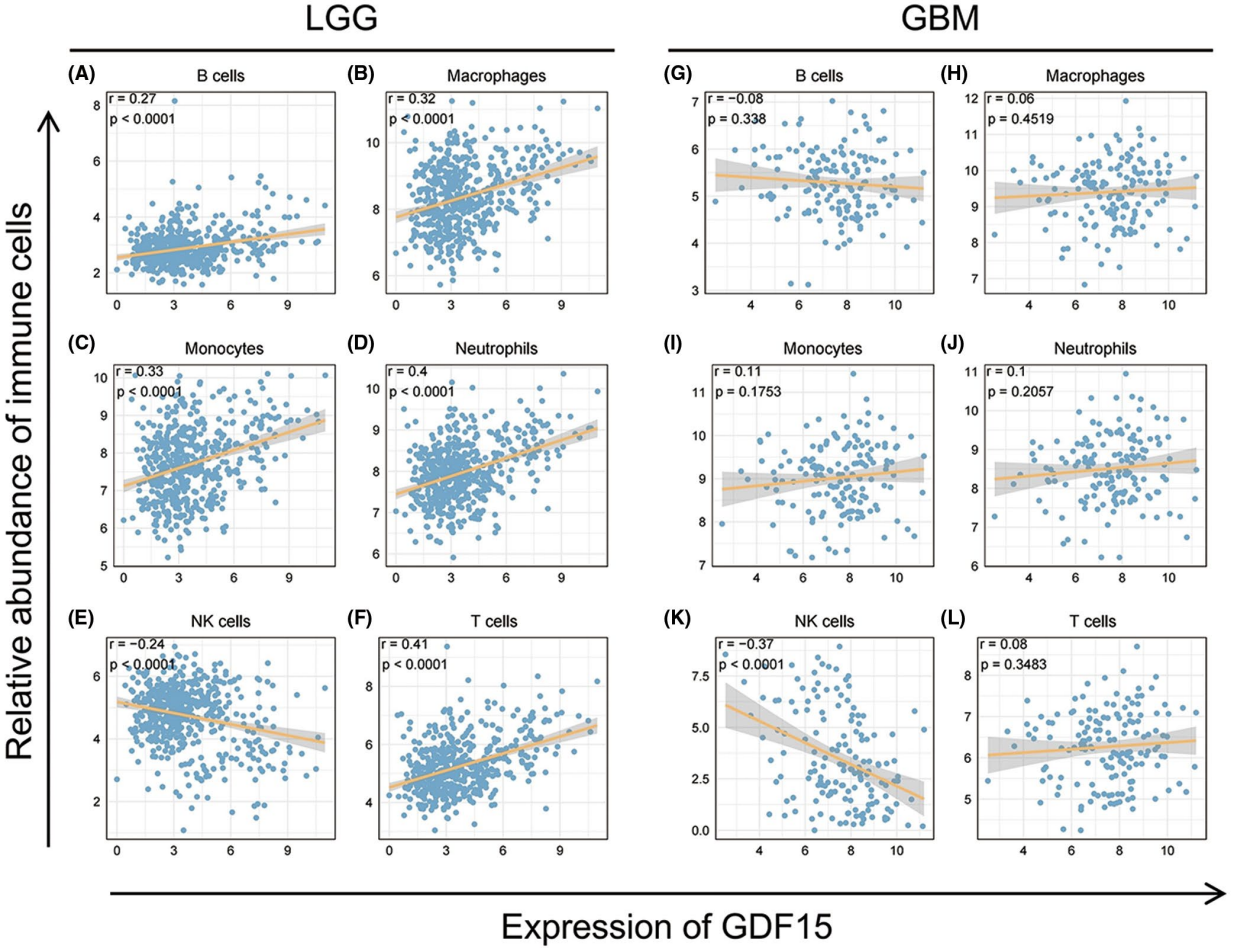
Correlation of GDF15 expression with relatively abundance of immune cells in LGG (A–F) and GBM (G–L) of TCGA dataset. Pearson's correlation test was used to calculate the correlation coefficient (r) and *p*‐value, and generated regression lines fitted to each dot plot

Tumor infiltrating immune cells constitute an important part of tumor microenvironment. In order to investigate the correlation between GDF15 and infiltrating immune cells, we used ImSig algorithm and six types of major infiltrating immune cells were selected, including B cells, T cells, macrophages, monocytes, neutrophils, and natural killer (NK) cells. As showed in Figure 4, GDF15 was significantly positively correlated with the relatively abundance of most of immune cells, including B cells, T cells, macrophages, monocytes, and neutrophils, in LGG of TCGA (Figure 4A–F). However, the links between GDF15 and these immune infiltrating cells had not been shown in GBM (Figure 4G–L). Only the relatively abundance of NK cells was negatively associated with GDF15 both in LGG and GBM. The results from CGGA showed a highly similar trend to LGG in TCGA (Figure S4A–F). Interestingly, GDF15 also showed remarkable association with immune infiltrating cells in GBM of CGGA (Figure S4G–L). This inconsistent trend was similar to our previous analysis of inflammatory response, suggesting there may be heterogeneity in microenvironment of GBM of two datasets. Considering the effect of tumor purity on microenvironment, we used TIMER algorithm to correct the expression level of key markers of immune cells for tumor purity, and then analyzed their correlation with GDF15 (Table 1). After adjusted by tumor purity, GDF15 also demonstrated positively correlation to key markers of B cells, T cells, macrophages, monocytes, and neutrophils in LGG, while most of the marker genes of NK cells were negatively correlated. GDF15 in GBM still did not show a clear connection with immune infiltrating cells. Collectively, these results revealed that GDF15 expression positively correlated with the infiltrating level of immune cells, except for NK cells, in LGG microenvironment.

**TABLE 1:** The correlation between GDF15 and gene markers of immune cells in TCGA dataset

Immune Cells	Gene markers	LGG				GBM			
		None		Purity		None		Purity	
		Cor	*p*‐value	Cor	*p*‐value	Cor	*p*‐value	Cor	*p*‐value
B cells	BLK	−0.005	0.916	−0.007	0.876	−0.078	0.360	−0.078	0.363
	CD19	0.194	***	0.199	***	−0.095	0.266	−0.096	0.264
	FCRL2	0.198	***	0.200	***	−0.010	0.911	−0.009	0.920
	MS4A1	0.270	***	0.273	***	−0.027	0.751	−0.027	0.756
	KIAA0125	0.175	***	0.178	***	−0.132	0.123	−0.134	0.118
	TNFRSF17	0.017	0.703	0.018	0.697	−0.217	*	−0.217	*
	TCL1A	0.151	***	0.153	***	−0.020	0.820	−0.019	0.826
	SPIB	0.285	***	0.286	***	0.015	0.858	0.016	0.850
	PNOC	−0.013	0.780	−0.012	0.792	−0.127	0.137	−0.128	0.136
T cells	CD6	0.349	***	0.380	***	0.033	0.697	0.040	0.645
	CD3D	0.414	***	0.429	***	0.004	0.967	0.006	0.942
	CD3E	0.461	***	0.473	***	0.011	0.894	0.014	0.870
	SH2D1A	0.305	***	0.328	***	0.057	0.508	0.061	0.481
	TRAT1	0.324	***	0.329	***	−0.014	0.874	−0.013	0.883
	CD3G	0.356	***	0.364	***	0.054	0.532	0.057	0.508
Macrophages	CD68	0.190	***	0.201	***	0.040	0.644	0.053	0.539
	CD84	0.026	0.576	0.029	0.531	−0.095	0.270	−0.103	0.231
	CD163	0.367	***	0.368	***	0.257	**	0.294	***
	MS4A4A	0.211	***	0.213	***	0.102	0.233	0.128	0.136
Monocytes	CSF1R	−0.145	**	−0.156	***	−0.020	0.812	−0.021	0.810
	KYNU	0.315	***	0.329	***	0.118	0.167	0.143	0.096
	PLA2G7	−0.069	0.133	−0.068	0.136	−0.142	0.096	−0.144	0.092
	ADAP2	0.072	0.118	0.078	0.088	−0.015	0.862	−0.014	0.868
	RASSF4	−0.262	***	−0.262	***	−0.188	*	−0.188	*
	FPR3	0.314	***	0.314	***	−0.033	0.699	−0.034	0.689
	TFEC	0.135	**	0.146	**	−0.089	0.299	−0.100	0.245
Neutrophils	FPR1	0.115	*	0.123	**	−0.014	0.873	−0.013	0.878
	SIGLEC5	0.182	***	0.192	***	0.046	0.594	0.050	0.563
	CSF3R	0.102	*	0.118	*	−0.025	0.772	−0.027	0.757
	FCAR	0.087	0.056	0.092	*	0.088	0.304	0.102	0.234
	FCGR3B	0.211	***	0.216	***	0.019	0.825	0.023	0.792
	CEACAM3	0.152	***	0.153	***	−0.043	0.621	−0.043	0.617
	S100A12	0.175	***	0.178	***	0.065	0.448	0.074	0.391
Natural killer cells	NCAM1	−0.369	***	−0.387	***	−0.292	***	−0.317	***
	KLRK1	−0.230	***	−0.237	***	−0.255	**	−0.255	**
	NCR1	0.152	***	0.152	***	−0.054	0.528	−0.054	0.532
	NCR2	−0.042	0.359	−0.042	0.360	−0.107	0.211	−0.108	0.208
	KLRD1	0.344	***	0.352	***	−0.039	0.647	−0.040	0.641
	KLRC2	−0.332	***	−0.346	***	−0.293	***	−0.293	***

Note

Cor, ρ value of Spearman's correlation. None, correlation without adjustment. Purity, correlation adjusted by tumor purity.

*p*‐value significant codes: 0 ≤ *** <0.001 ≤ ** <0.01 ≤ * <0.01.

### 3.5 GDF15 positively associated with immune checkpoint molecules in glioma

Expression and regulation of immune checkpoint molecules play crucial role in the response to immunotherapy. Therefore, we analyzed the relationship between GDF15 and several immune checkpoint molecules that had been studied in‐depth, including PD‐1 (PDCD1), PD‐L1 (CD274), PD‐L2 (PDCD1LG2), CTLA‐4 (CTLA4), LAG‐3 (LAG3), TIM‐3 (HAVCR2), IDO (IDO1), CD40, OX40 (TNFRSF4), GITR (TNFRSF18), and ICOS. We found that GDF15 was positively associated with all immune checkpoint molecules in LGG of TCGA, of which PD‐1, IDO, OX40, and ICOS showed a relatively strong correlation (Table 2, Figure S5A). However, only PD‐L1, PD‐L2, IDO, OX40, and GITR were significantly correlated to GDF15 in GBM (Figure S5B). Similar results were observed in LGG of CGGA (Table 2, Figure S5C). Specifically, the expression level of GDF15 in CGGA‐GBM also had a positive correlation with all immune checkpoint molecules, which was different from TCGA (Figure S5D). Together with the results of immune infiltration analysis, GDF15 may play its role in regulating the immune microenvironment more in LGG, while the effect was not remarkable in GBM.

**TABLE 2:** Association between GDF15 and immune checkpoint molecules in glioma

Markers	Genes	TCGA				CGGA			
		LGG		GBM		LGG		GBM	
		Correlation	*p*‐value	Correlation	*p*‐value	Correlation	*p*‐value	Correlation	*p*‐value
PD−1	PDCD1	0.49	**<0.001**	0.12	0.147	0.35	**<0.001**	0.45	**<0.001**
PD‐L1	CD274	0.32	**<0.001**	0.36	**<0.001**	0.34	**<0.001**	0.53	**<0.001**
PD‐L2	PDCD1LG2	0.32	**<0.001**	0.17	**0.041**	0.38	**<0.001**	0.55	**<0.001**
CTLA−4	CTLA4	0.23	**<0.001**	0.01	0.914	0.21	**<0.001**	0.30	**<0.001**
LAG−3	LAG3	0.20	**<0.001**	−0.06	0.451	0.23	**<0.001**	0.24	**<0.001**
TIM−3	HAVCR2	0.18	**<0.001**	−0.06	0.436	0.32	**<0.001**	0.43	**<0.001**
IDO	IDO1	0.45	**<0.001**	0.27	**<0.001**	0.40	**<0.001**	0.28	**<0.001**
CD40	CD40	0.35	**<0.001**	0.16	0.054	0.56	**<0.001**	0.56	**<0.001**
OX40	TNFRSF4	0.45	**<0.001**	0.25	**0.002**	0.41	**<0.001**	0.38	**<0.001**
GITR	TNFRSF18	0.28	**<0.001**	0.29	**<0.001**	0.21	**<0.001**	0.37	**<0.001**
ICOS	ICOS	0.44	**<0.001**	0.11	0.185	0.33	**<0.001**	0.27	**<0.001**

Note

Bold values indicate *p*‐value <0.05.

### 3.6 GDF15 predicted poor survival in lower grade glioma

**FIGURE 5:**
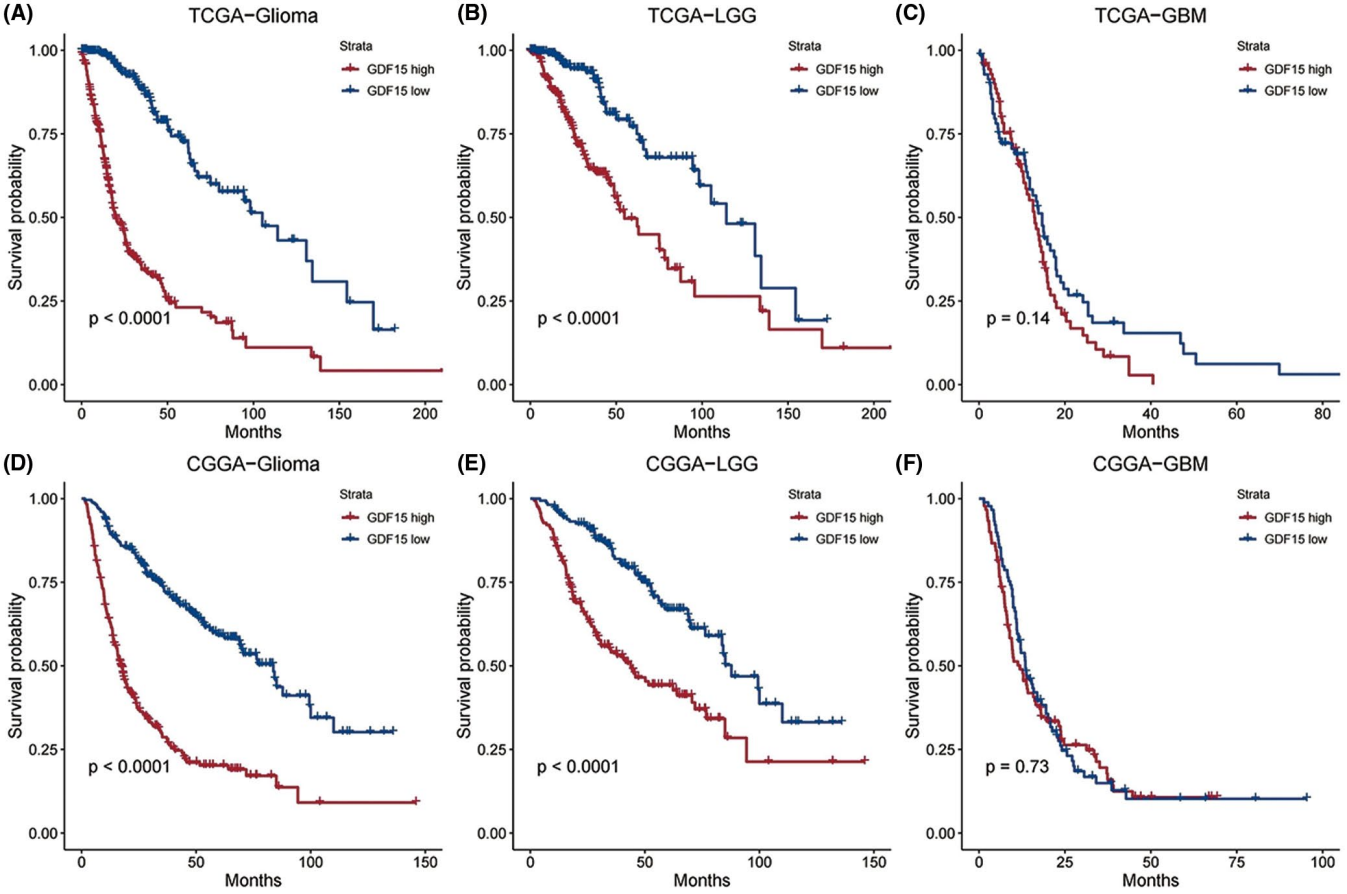
Kaplan–Meier survival analysis of glioma based on GDF15 expression. Data from TCGA dataset showed high expression level of GDF15 predicted poor survival in entire glioma (A) and LGG (B), while no statistical difference was found in GBM (C). Similar results were verified in entire glioma (D), LGG (E), and GBM (F) patients of CGGA dataset, respectively

To explore the prognostic value of GDF15 in glioma, Kaplan–Meier survival analysis were performed. We found that high expression level of GDF15 was significantly associated with worse overall survival (OS) of glioma both in TCGA (*p* < 0.0001) and CGGA (*p* < 0.0001) (Figure 5A,D). The same association was observed while analyzing only the LGG with *p* < 0.0001 and *p* < 0.0001 for TCGA and CGGA, respectively (Figure 5B,E). However, no significant correlation was found in OS of GBM in TCGA (*p* = 0.14) or CGGA (*p* = 0.73) (Figure 5C,F). Subsequently, we analyzed the effect of GDF15 on progression‐free survival (PFS) of glioma in TCGA (Figure S6A–C). The results were similar to the analysis of OS, and glioma with low GDF15 expression have better PFS (*p* < 0.0001). Subgroup analysis revealed the significance of PFS was only showed in LGG (*p* = 0.00065), while no significant difference was observed in GBM (*p* = 0.06).

Univariate and multivariate Cox regression analysis for LGG showed that GDF15, grade, and 1p19q status were significantly related to prognosis both in two datasets (Table 3). Since the *p*‐value (*p* = 0.058) of the correlation between IDH status and prognosis in CGGA was close to the statistical threshold, combined with its important biological significance, it also needed to be considered as a prognostic factor. No significant association between GDF15 and OS was found in subsequent Cox analysis for GBM (Table S2). Taken together, these results revealed that GDF15 could be served as interesting prognostic biomarker for LGG.

**TABLE 3:** Univariate and multivariate Cox regression analyses of various clinical and molecular features related to survival in LGG

Characteristics	TCGA‐LGG						CGGA‐LGG					
	Univariate			Multivariate			Univariate			Multivariate		
	HR	95% CI	*p*‐value	HR	95% CI	*p*‐value	HR	95% CI	*p*‐value	HR	95% CI	*p*‐value
GDF15	1.53	1.38–1.69	**<0.001**	1.2	1.05–1.37	**0.006**	1.36	1.22–1.51	**<0.001**	1.23	1.08–1.4	**0.002**
Age	1.06	1.04–1.08	**<0.001**	1.06	1.04–1.07	**<0.001**	1.01	0.99–1.03	0.235	1.01	1–1.03	0.17
Gender	1.01	0.68–1.5	0.953	1.05	0.69–1.58	0.822	1.26	0.89–1.78	0.197	1.42	0.99–2.06	0.06
Grade	2.95	1.91–4.55	**<0.001**	1.64	1.02–2.62	**0.04**	2.85	1.93–4.21	**<0.001**	2.93	1.96–4.39	**<0.001**
IDH status	6.64	4.4–10	**<0.001**	2.1	1.13–3.89	**0.019**	2.21	1.54–3.18	**<0.001**	1.56	0.99–2.46	0.058
1p19q status	2.51	1.52–4.14	**<0.001**	2.2	1.25–3.88	**0.006**	2.63	1.69–4.09	**<0.001**	1.99	1.23–3.2	**0.005**

Abbreviations: HR, Hazard ratio. CI, Confidence interval.

Bold values indicate *p*‐value <0.05.

### 3.7 Construction and validation of a GDF15‐related prognostic nomogram

**FIGURE 6:**
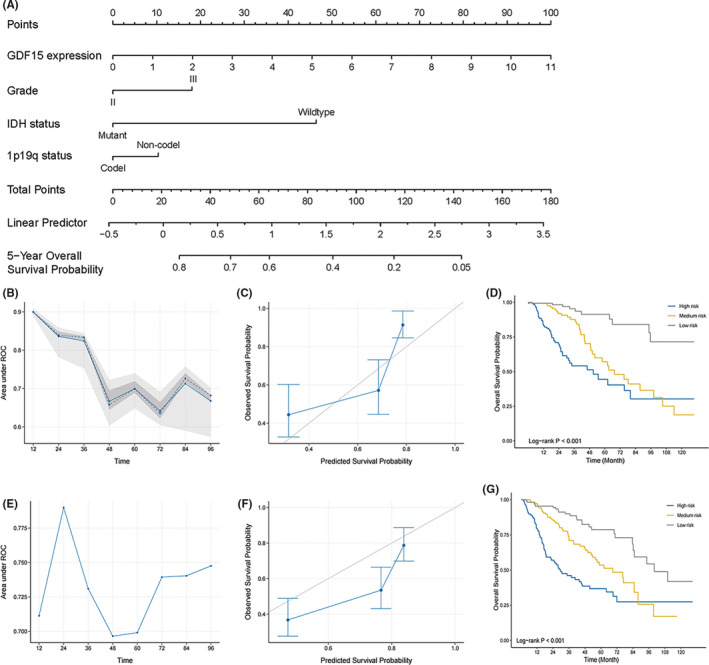
Construction and validation of a GDF15‐related prognostic nomogram. (A) Nomogram for predicting survival in LGG patients. The top row showed the point value for each variable. The point value of each variable was summarized to total points and determined the likelihood of 5 years survival. (B–G) Time‐dependent AUC showed the predictive performance of model in different time period in primary (B) and validation (E) cohorts. The calibration curve displayed the difference between the prediction of model of 5 years survival and the actual survival outcome in primary (C) and validation (F) cohorts. The risk classification system based on nomogram divided LGG patients into three risk groups and showed distinct survival curves in primary (D) and validation (G) cohorts

For the sake of taking advantage of the prognostic value of GDF15 in LGG, we constructed a GDF15‐related nomogram and risk classification system for predicting survival. The cases from TCGA were used as primary cohort to construct the predictive model, and the cases from CGGA were chosen as validation cohort for verification of the model. Based on above results, the prognostic nomogram model was established (Figure 6A). The model performance was assessed internally by time‐dependent AUC (area under the receiver operating characteristic curve) (Figure 6B). In the prediction of early survival rate (<3 years), the predictive ability of model was exceptional, and the AUC was all greater than 0.8. Over time, the AUC declined slightly but still remained at around 0.7. The calibration plot for the probability of survival at 5 years showed an admirable agreement between the prediction and observation in primary cohort (Figure 6C). A risk classification system was subsequently developed based on the nomogram. After dividing the primary cohort into three risk groups, it can be seen from the Kaplan–Meier survival curves that the prognosis of low‐risk group was significantly better than that of medium and high‐risk groups (log‐rank *p* < 0.001) (Figure 6D). Similar to primary cohort, the predictive model could predict survival well in the validation cohort, and the AUC remains above 0.7 in all time periods (Figure 6E). The calibration plot for validation cohort showed a valuable agreement between the prediction and observation in 5 years survival (Figure 6F). Low‐risk group of validation could also achieve favorable OS compared to medium and high‐risk groups (log‐rank *p* < 0.001) (Figure 6G). Collectively, these findings further proved the prognostic value of GDF15, and combined with other prognostic factors could better predict the prognosis of LGG.

## 4 DISCUSSION

Traditional treatment for glioma did not achieve a breakthrough development in last decade. Although the optimization of traditional treatment showed certain advantages, further relevant clinical researches are needed for verification.^24^, ^25^, ^26^ As one of the most promising approaches in oncology field, immunotherapy have shown encouraging success for various types of cancer treatment.^3^ However, the current prospects of immunotherapy for glioma are not optimistic.^4^, ^5^, ^6^ The hindrances to make immune checkpoint inhibitors effectively therapeutic in glioma include a low immunogenic response and extensive intratumoral heterogeneity.^6^, ^27^, ^28^ These challenges spur us to explore additional biomarkers which may regulate the immune microenvironment and improve efficacy of immunotherapy for brain tumors. In this study, we suggested that GDF15 play an important role in malignant progression of glioma and the immune microenvironment, and could be served as a novel prognostic and immune related biomarker.

GDF15 has been demonstrated its multiple important roles in several diseases comprising obesity, cachexia, and cardiovascular disease.^7^, ^8^, ^9^ With the deepening of research, GDF15 has been shown to be closely related to the occurrence and development of some types of tumors.^11^, ^12^, ^13^ For glioma, several studies have shown that GDF15 is associated with many cellular mechanisms, such as apoptosis, migration, and invasion.^16^, ^29^, ^30^ Although GDF15 is closely related to the immune system and is considered to be the center of inflammation‐induced tissue tolerance,^10^ there are few studies on the role of GDF15 in tumor immune regulation. Roth et al showed that GDF15 may affect the susceptibility of glioma cells toward natural killer cells and splenocytes, thus contributing to the proliferation and immune escape of malignant glioma.^31^ However, currently, there has not yet been an article comprehensively analyzing the specific clinical and immune features of GDF15 in glioma.

In this study, we first systematically explored the expression of GDF15 in various types of glioma. We found that the expression level of GDF15 was positively correlated with tumor grade and other markers indicated poor prognosis, such as IDH, 1p19q, EGFR, PTEN, and chromosomes 7 and 10. The results of GSEA and GSVA analysis revealed GDF15 was significantly correlated with several important immunity process, such as inflammatory response, TNFɑ signaling via NF‐κB, and interferon response. Mechanistically, signaling through transforming growth factor β receptor I and activating Smad1/5, GDF15 may reduce the expression of IL‐12 receptor β2 expression on NK cells and in turn regulated interferon gamma response.^32^ By inhibiting TGF‐β‐activated kinase (TAK1) signaling to NF‐κB, GDF15 could suppress the activity of macrophage, thereby blocking synthesis of TNF.^33^ Besides, our results showed that GDF15 was related to angiogenesis, implying that it may affect tumor progression. The experiments in vitro and verified our findings. Down‐regulation of GDF15 led to a decrease in the expression of NF‐κB pathway and tumor invasion‐related proteins. Meanwhile, migration ability of glioma cell lines was apparently suppressed. The effect of GDF15 on the invasion and migration of glioma cells indicated its key role in tumor progression.

Another important finding of our research was that GDF15 closely correlated to infiltrating immune cells and immune checkpoint molecules, especially in LGG. We found that GDF15 was positively associated with the relatively abundance of most of immune cells in LGG, while only negatively correlated to NK cells. The effect of GDF15 on NK cells was different from other immune infiltrating cells, which was mainly due to GDF15 down‐regulated the expression of IL‐12 receptor β2 on NK cells, thereby promoting NK cell dysfunction.^32^ In addition, GDF15 was positively associated with major immune checkpoint molecules in LGG. However, the results of correlation analysis among GDF15, infiltrating immune cells, and immune checkpoint molecules in GBM from TCGA and CGGA were inconsistent. We cautiously believe that the significant heterogeneity of tumor genetics and immune microenvironment in GBM may be the main reason.^6^, ^34^ In fact, even individual cells in glioblastoma differed in the expression of oncogenic transcriptional programs.^28^ In addition, the glioma gene expression data collected by the TCGA and CGGA datasets come from different regions, and the ethnic composition is quite different, which may further deepen the heterogeneity between GBM populations. However, there was no relevant research to analyze the difference of immune landscape in GBM between TCGA and CGGA. We would conduct deeper research in the future to verify our views.

In recent years, improvements in sequencing technology were facilitating increasingly deep studies of glioma. Researchers have reported that many genetic biomarkers, such as CAMKK2,^35^ GINS2,^36^ FOXD1‐AS1,^37^ CpGs methylation,^38^ TRIB2, and MAP3K1,^39^ have significant impact on the prognosis of glioma and could be used as prognosis predictors of glioma. These predictors were of great significance for in‐depth understanding of the disease mechanism of glioma. In our study, Kaplan–Meier survival analysis and COX regression analysis revealed that high GDF15 expression level predicted poor overall and progression‐free survival in LGG, highlighting its potential to be served as a novel prognostic biomarker. Based on these findings, we established a GDF15‐related nomogram predictive model and divided LGG into different risk groups. For high‐risk group, more aggressive treatment may help prolong its unfavorable prognosis. On the contrary, the low‐risk group may consider receiving more conservative treatment to improve the overall quality of life.

## 5 CONCLUSIONS

In conclusion, this study confirmed the important role of GDF15 for the malignant progression and immune microenvironment of glioma, especially in LGG. Regulating the expression of GDF15 may help solve the dilemma of immunotherapy in glioma. GDF15 was significantly correlated to poor survival of LGG, suggesting its potential to be served as a novel prognostic biomarker.

## CONFLICT OF INTEREST

The authors declare no conflict of interest.

## AUTHOR CONTRIBUTIONS

S Du, C Ren and L Guo conceived and designed the study. L Guo, N Tang, R Liu and Y Qin performed data collection and assembly. Y Chen, S Hu, and L Gao performed experiments in vitro. L Guo and Y Chen analyzed, interpreted and visualized data, and were major contributors in writing the manuscript. All authors read and approved the final manuscript.

## Supporting information

Fig S1

Fig S2

Fig S3

Fig S4

Fig S5

Fig S6

Table S1

Table S2

Table S3

Supplementary Material

## Data Availability

TCGA dataset is available at GlioVis (http://gliovis.bioinfo.cnio.es/), and CGGA dataset is available at CGGA (http://www.cgga.org.cn/). Web tools and R packages used in this study are included in article.
