# Supplementary figures and images for: GDF15 expression in glioma is associated with malignant progression, immune microenvironment, and serves as a prognostic factor
Source: CNS Neurosci Ther. 2021 Oct 25;28(1):158–71. doi: 10.1111/cns.13749 (PMC8673705; doi:10.1111/cns.13749)

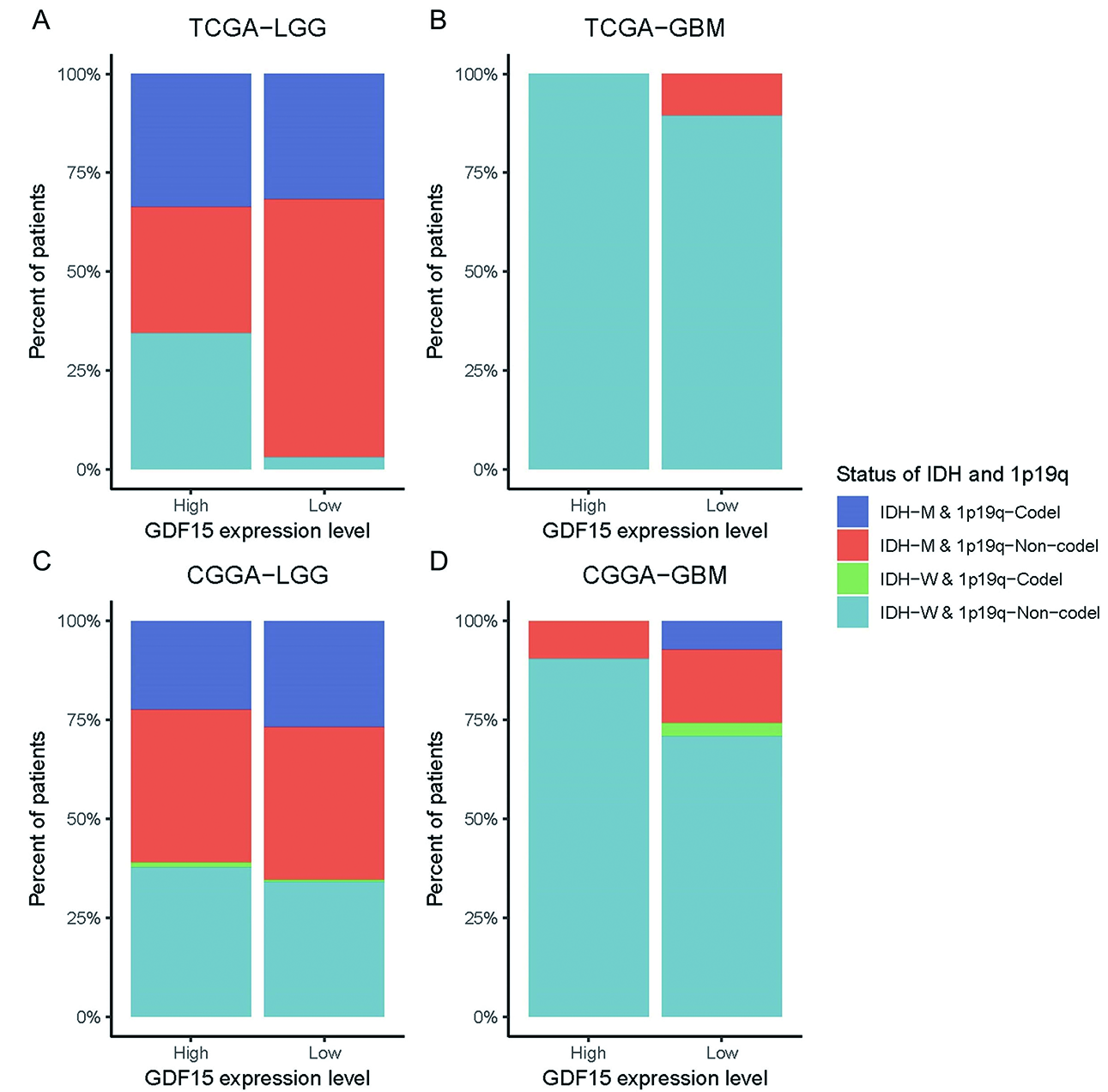

Supplement: Supplementary file 1 — Fig S1 [file CNS-28-158-s005.tif]

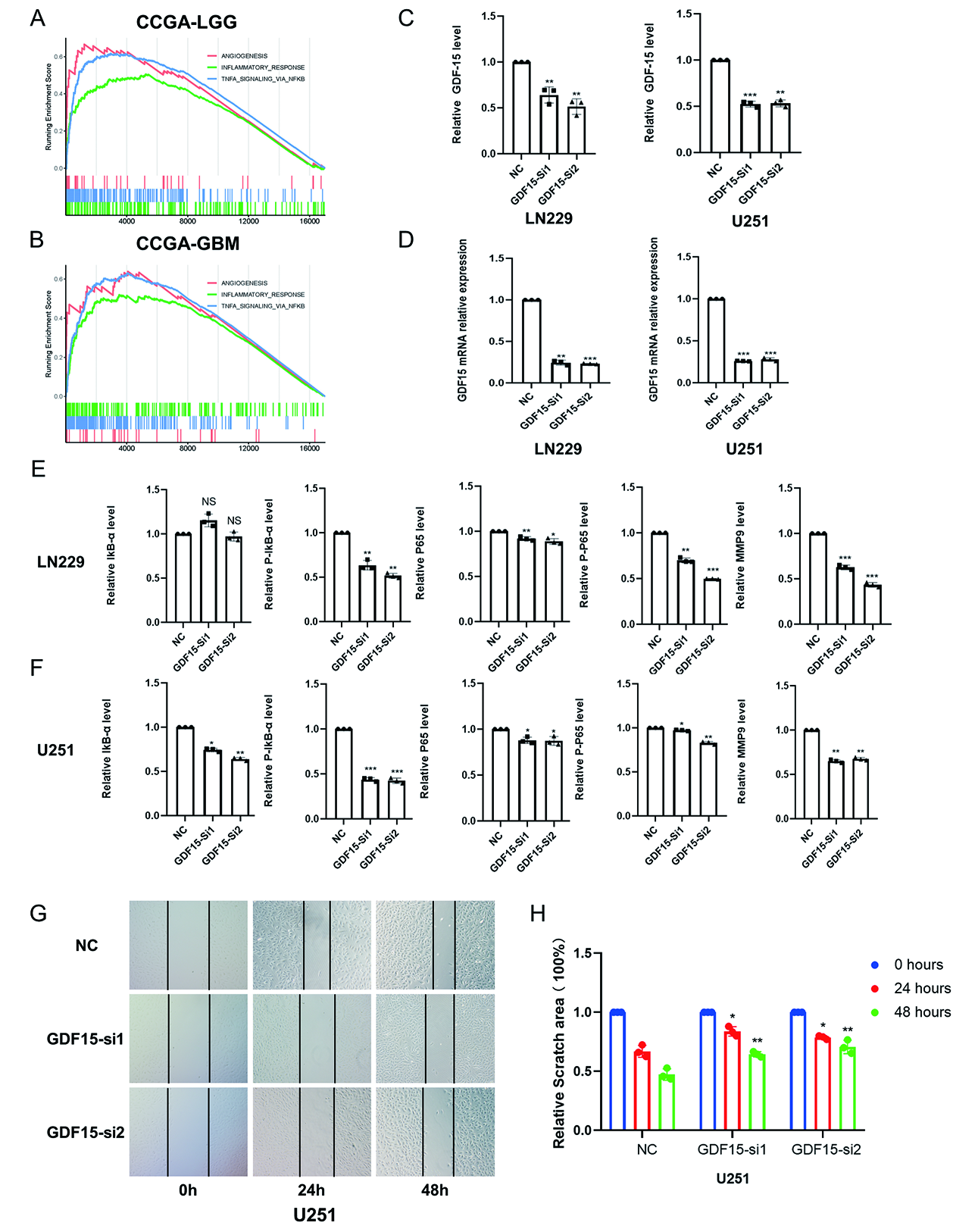

Supplement: Supplementary file 2 — Fig S2 [file CNS-28-158-s004.tif]

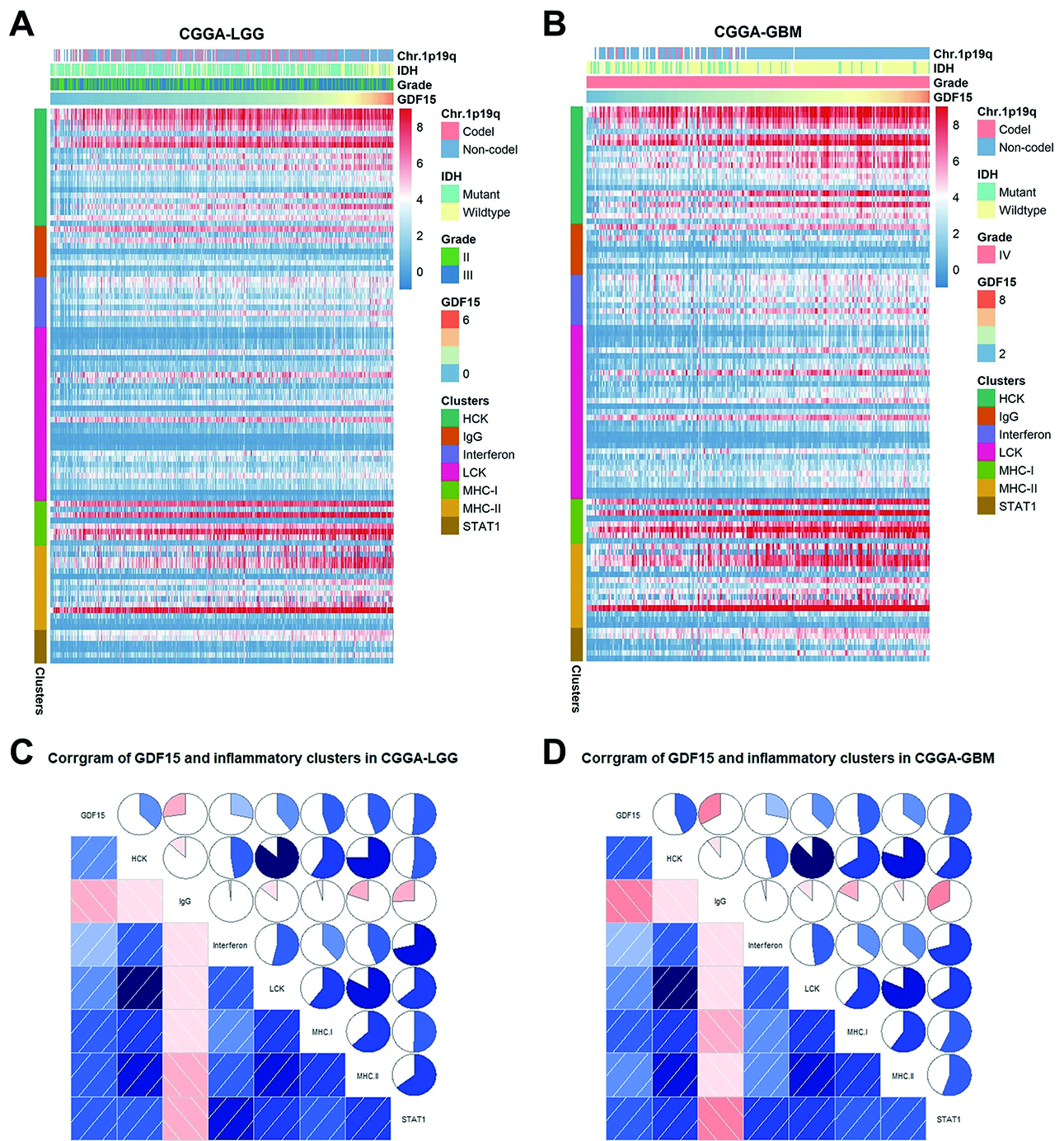

Supplement: Supplementary file 3 — Fig S3 [file CNS-28-158-s003.tif]

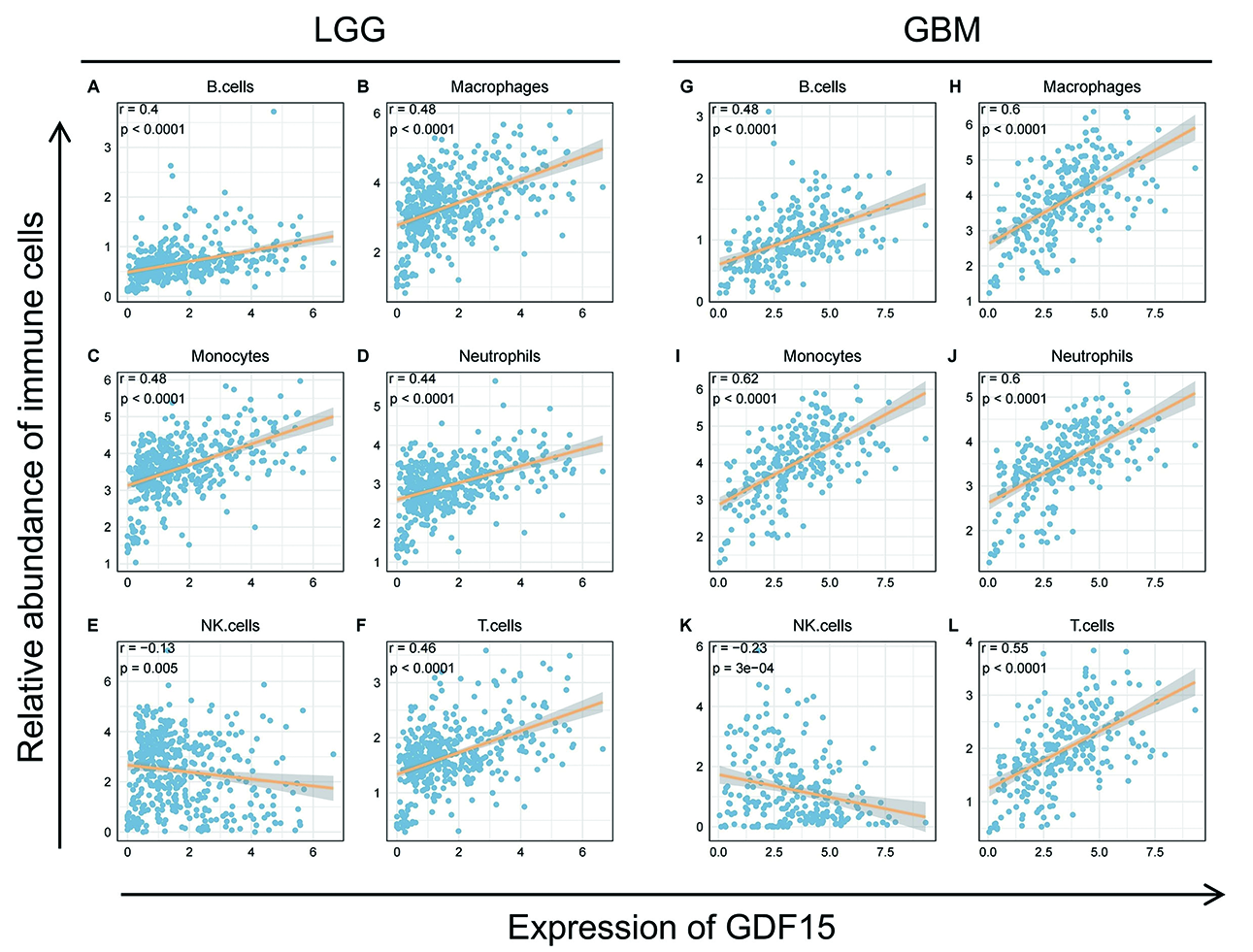

Supplement: Supplementary file 4 — Fig S4 [file CNS-28-158-s008.tif]

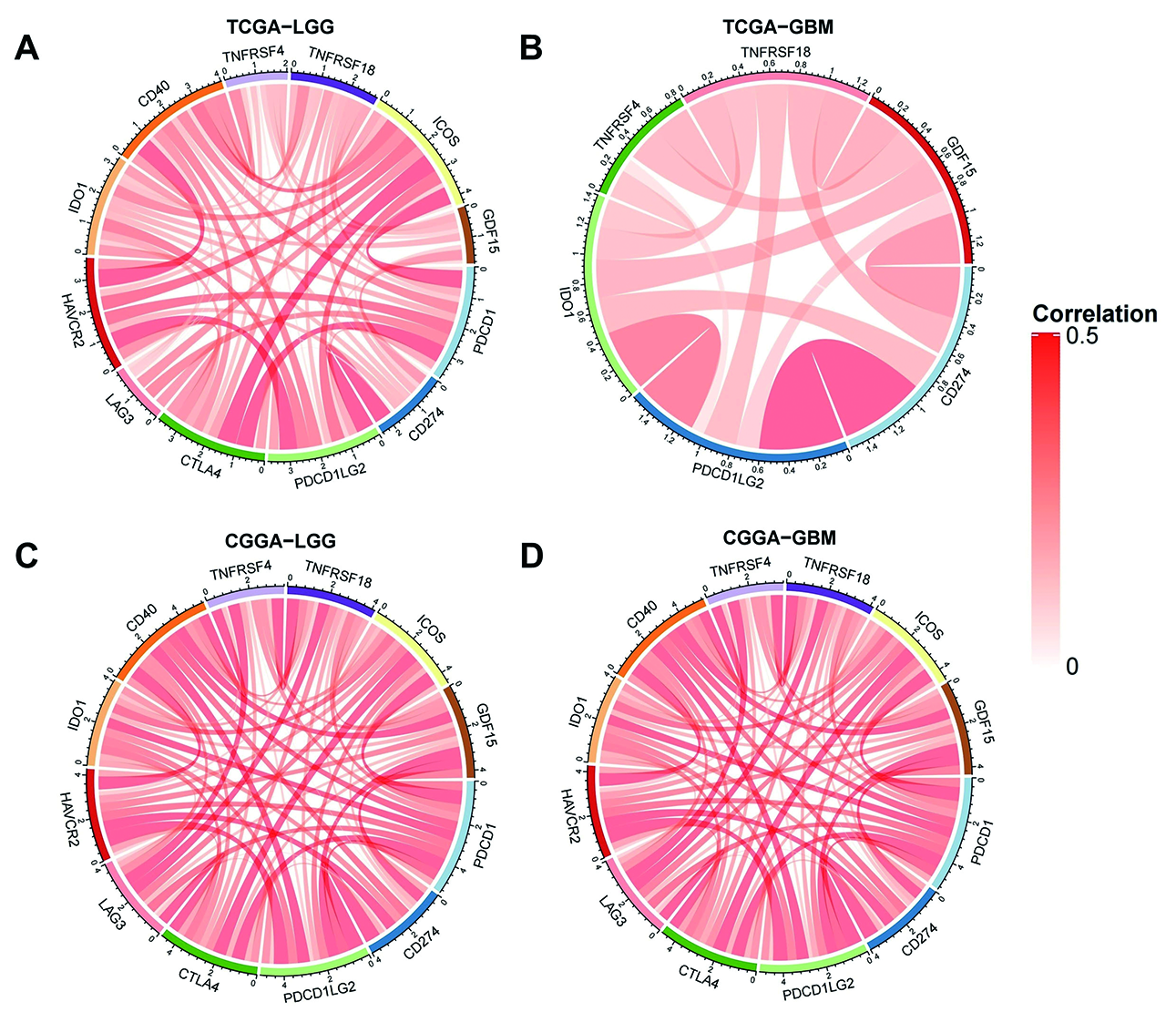

Supplement: Supplementary file 5 — Fig S5 [file CNS-28-158-s006.tif]

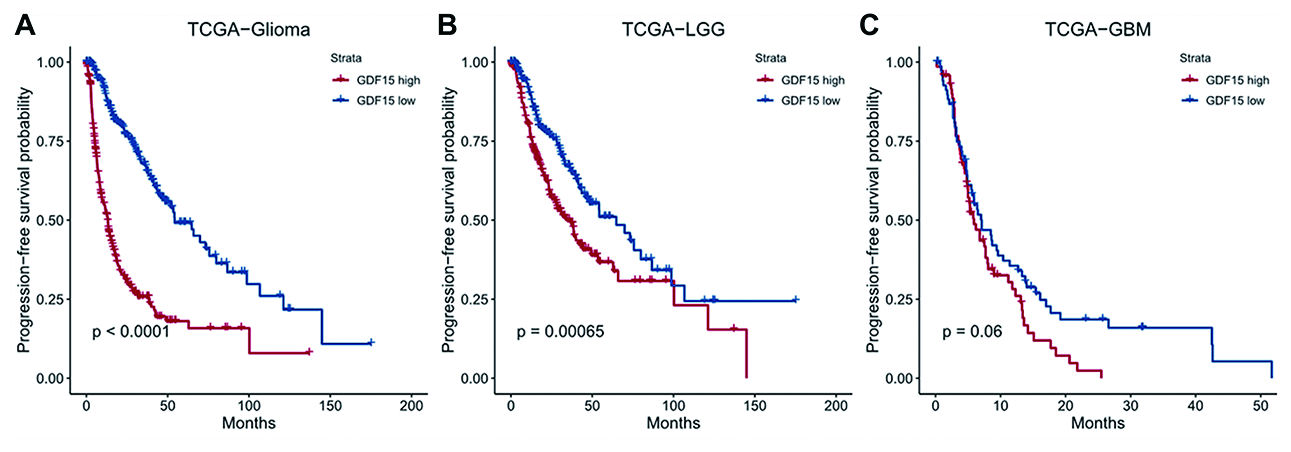

Supplement: Supplementary file 6 — Fig S6 [file CNS-28-158-s009.tif]
