# Supplementary material for: GDF15 expression in glioma is associated with malignant progression, immune microenvironment, and serves as a prognostic factor
Source: CNS Neurosci Ther. 2021 Oct 25;28(1):158–71. doi: 10.1111/cns.13749 (PMC8673705; doi:10.1111/cns.13749)
Supplement: Supplementary file 10 — Supplementary Material [file CNS-28-158-s002.docx]

Figure S1. The proportion distribution of patients with different status of IDH and 1p19q in high- and low-GDF15 expression groups in LGG (A) and GBM (B) of TCGA and in LGG (C) and GBM (D) of CGGA. M, mutant. W, wildtype. Codel, co-deletion. Non-codel, non-co-deletion.

Figure S2. Biological function investigation and in vitro verification of GDF15 in glioma. (A, B) GSEA analysis confirmed several important functions of GDF15 in LGG (A) and GBM (B) of CGGA datasets. (C) Quantification of immunoblot results of GDF15 mRNA expression in LN229 and U251 cell lines after infection with GDF15 siRNA or negative control. (D) The real-time quantitative PCR assay of GDF15 mRNA expression in LN229 and U251 cell lines after infection with GDF15 siRNA or negative control. (E, F) Quantification of the Western blot of NF-κB signaling and MMP9 protein expression in LN229 (E) and U251 (F) cell lines after transfection with GDF15 siRNA or negative control. (G) Cell scratch assay of U251 cell lines treated with GDF15 siRNA or negative control (magnification, ×100). (H) Quantification of cell scratch assay of U251 cell lines.

Figure S3. Relationship between GDF15 and inflammatory response in glioma of TCGA dataset. (A, B) Heatmaps showed the disturbance of clinicopathological parameters, GDF15 expression, and seven metagene clusters in LGG (A) and GBM (B) of CGGA datasets. (C, D) Corrgrams were generated according to Pearson’s correlation values based on GDF15 expression and GSVA enrichment scores for seven metagene clusters. Blue and red represented positive and negative correlations, respectively. Darker colors indicated more significant correlations.

Figure S4. Correlation of GDF15 expression with relatively abundance of immune cells in LGG (A–F) and GBM (G–L) from CGGA. Each dot represented a single glioma patient. Pearson’s correlation test was used to calculate the correlation coefficient (r) and *P*-value (significance level), and generated regression lines fitted to each dot plot.

Figure S5. Chord diagrams showed the correlation between GDF15 and immune checkpoint molecules in TCGA (A, B) and CGGA (C, D) datasets.

Figure S6. Kaplan–Meier progression-free survival analysis of glioma based on GDF15 expression. Data from TCGA dataset showed high expression level of GDF15 predicted poor progression-free survival in entire glioma (A) and LGG (B), while no statistical difference was found in GBM (C).
